# Starfield’s 4Cs of NCD management in primary healthcare: a conceptual framework development from a case study of 19 countries

**DOI:** 10.1136/bmjgh-2024-017578

**Published:** 2025-01-29

**Authors:** Chuan De Foo, Krishaa Logan, Elliot Eu, Darius Erlangga, Juan Carlos Rivillas, Ewa Kosycarz, Aungsumalee Pholpark, Natchaya Ritthisirikul, Piya Hanvoravongchai, Likke Prawidya Putri, Tiara Marthias, Marcela Schenck, Wilson Benia, Eva Turk, Kim Bao Giang, Doan Thi Thuy Duong, Supri Shrestha, Maria Eugenia Esandi, Laura Antonietti, Shangzhi Xiong, Pami Shrestha, Jasper Tromp, Helena Legido-Quigley

**Affiliations:** 1 Saw Swee Hock School of Public Health, National University Singapore and National University Health System, Singapore; 2 Duke-NUS Medical School, Singapore; 3 Occupational and Environmental Medicine Department, Singapore General Hospital, Singapore; 4 Global Health and Development, London School of Hygiene & Tropical Medicine, London, UK; 5 School of Public Health, Imperial College London, London, UK; 6 SGH Warsaw School of Economics, Warsaw, Poland; 7 Faculty of Social Sciences and Humanities, Mahidol University, Salaya, Nakhon Pathom, Thailand; 8 Thailand National Health Foundation, Bangkok, Thailand; 9 Faculty of Medicine, Chulalongkorn University, Bangkok, Thailand; 10 Gadjah Mada University Center for Health Policy and Management, Yogyakarta, Indonesia; 11 Nossal Institute for Global Health, University of Melbourne, Melbourne, Victoria, Australia; 12 Universidad de la República, Montevideo, Uruguay; 13 Pan American Health Organization, Washington, Washington, USA; 14 Centre for Digital Health and Social Innovation, University of Applied Sciences, St. Pölten, Austria; 15 University of Maribor, Maribor, Slovenia; 16 School of Preventive Medicine and Public Health, Hanoi Medical University, Hanoi, Viet Nam; 17 Hanoi University of Public Health, Hanoi, Viet Nam; 18 Nepal Medical College Teaching Hopsital, Kathmandu, Nepal; 19 Departamento de Economía, Universidad Nacional del Sur, Bahia Blanca, Argentina; 20 Universidad Nacional Arturo Jauretche, Florencio Varela, Buenos Aires, Argentina; 21 Faculty of Medicine and Health, The George Institute for Global Health and University of New South Wales, Sydney, NSW, Australia; 22 The George Institute for Global Health, London, UK

**Keywords:** health policies and all other topics, public Health, global health, health services research, health systems

## Abstract

**Introduction:**

Faced with a backdrop of an increasing chronic disease burden from an ageing global population compounded with rising healthcare costs, health systems are required to implement cost-effective, safe and equitable care through efficient service delivery models. One approach to achieving this is through Starfield’s 4Cs of primary healthcare (PHC), which delineates the key attributes of a high-performing PHC system that upholds the pillars of care coordination, first contact of care, continuity of care and comprehensive care. Therefore, this study aims to explore and elucidate the key themes and subthemes related to and extending beyond Starfield’s 4Cs of PHC by integrating findings from a comprehensive literature review and a qualitative study.

**Methods:**

In this case study analysis, case studies of PHC systems from 19 countries were purposefully selected to represent a range of income levels and diversity in health systems and PHC landscapes. A review of existing literature of peer-reviewed articles, policy documents and technical reports made publicly available data on PHC was complemented with data obtained from 61 in-depth interviews with health systems experts from a larger study. The research team thematically analysed the data and organised the key themes and subthemes into a conceptual framework that is anchored on Starfield’s 4Cs of PHC.

**Results:**

Broadly, we developed a conceptual framework with the 4Cs, placing providers and patients at the centre. The key subthemes that manifested from Starfield’s 4Cs included maximising the use of existing fiscal resources, leveraging technology, improving accessibility to health services and task sharing. Other relevant and overarching themes were the deployment of national frameworks, equity, healthcare provider retention, service integration, emergency preparedness and community engagement.

**Discussion:**

The subthemes derived point health systems in the right direction based on the trialled and tested PHC models of various countries. Their strong points were highlighted in our case studies to depict how Starfield’s 4Cs are leveraged to strengthen PHC, and the themes we identified that went beyond the 4Cs are necessary considerations for modifying PHC policies going forward.

**Conclusion:**

As the world enters an era of ageing populations and acute system shocks, PHC needs to be fortified and integrated into the more extensive system to protect the health of the population and safeguard the well-being of providers. Our conceptual framework offers health systems a glimpse of how this can be achieved.

**WHAT IS ALREADY KNOWN ON THIS TOPIC**

- Primary healthcare (PHC) has been established as the foundation of an effective health system, to provide accessible and affordable care in the face of the ageing population and the growing disease burden of non-communicable chronic diseases (NCDs).
- A robust PHC system embodies Starfield’s 4Cs, namely care coordination, first contact of care, continuity of care and comprehensive care.
- It is established that a PHC system that achieves these 4Cs is associated with better-quality services, lower healthcare costs, reduced inequality and better population health.

**WHAT THIS STUDY ADDS**

- Through our review of PHC systems in 19 countries, we derived a comprehensive framework that identifies several subthemes within and beyond Starfield’s 4Cs of PHC, generating elements of a successful people-centred and integrated PHC system.
- Our research also highlights the challenges and limitations that countries face in achieving the 4Cs, including fiscal constraints, human resource availabilities and barriers to equitable healthcare accessibility.

**HOW THIS STUDY MIGHT AFFECT RESEARCH, PRACTICE OR POLICY**

- Strengthening PHC systems through tailoring the subthemes of the 4Cs for a fit-for-purpose approach is essential in managing the rise in NCD burden, particularly in the context of postpandemic healthcare challenges.
- The delivery of sustainable and equitable care for patients with NCD requires prioritisation of PHC development as a long-term, cost-effective strategy, reinforced by co-engineered care models that foster collaboration across care interfaces, community partners and multiple levels of healthcare.

## Introduction

 The 1978 Alma-Ata Declaration defined primary healthcare (PHC) as the cornerstone of an effective health system and declared it a global priority.^1^ PHC should be the first level of care that takes a whole-of-society approach that offers services closer to the communities where people present their health problems and most curative and preventive health needs are satisfied. In 2018, global leaders renewed their commitment to PHC by ratifying the Declaration of Astana. It emphasised the need to build a sustainable PHC system by providing accessible and affordable delivery models and empowering individuals and communities to drive their care.^2^ In practice, the key tenets from the declaration are unchanged but are implemented differently across regions and countries due to prevailing political, economic, cultural and social factors that influence how PHC is delivered in every country.^3^

A strong PHC system is expected to tackle the various challenges of an ageing population and widening health inequities. Transformations at the PHC interface, from multidisciplinary teams to innovative models of financing that span across the different levels of care, are the key to managing multimorbidity more optimally and controlling increasing healthcare costs.^4^ From a health equity perspective, PHC can reach ‘neglected’ communities when aided by community health workers who may extend basic health services to these communities. For example, in countries such as Brazil, India, Thailand and Canada, village health workers (VHWs) played a pivotal role during the COVID-19 pandemic by conducting surveillance, delivering medicines and engaging in public educational activities. These efforts continued postpandemic to address broader healthcare needs.^5^

Unfortunately, as the COVID-19 pandemic captured the world’s attention and stretched healthcare resources over the past years, non-communicable chronic diseases (NCDs) such as hypertension, heart disease and diabetes have remained the leading causes of mortality worldwide.^6^ The shifting of healthcare resources to tackle the pandemic has created a backlog of NCD and other non-COVID-19-related service obligations. Therefore, PHC must be placed at the top of the political agenda as health systems undergo transformative strengthening and as the world slowly clears the backlog of NCD service obligations, although mainly through tertiary care. Concomitantly, PHC can be harnessed as a strategy for health systems to optimise the deployment of scarce resources. In the face of a slowing global economy, competing priorities for healthcare financing and rising healthcare expenditures due to the growing NCD burden, PHC should be upheld as a key means for the delivery of quality and efficient care.^7^

However, despite countries having long stated their commitment to improving PHC, three-quarters of people in the world—including at least 85% of people in low- and middle-income countries (LMICs)—still do not benefit from accessible, affordable and effective PHC.^8^ Narrowing this gap has been a tall order for most health systems. The foundation of a robust health system is a strong PHC interface.^9^ It can be delineated as a system that upholds Starfield’s 4Cs of PHC, encompassing the pillars of care coordination, first contact of care, continuity of care and comprehensive care. There is general agreement in the context of PHC that achieving these 4Cs is associated with better quality services, lower healthcare costs, reduced inequality and better population health.^10 11^

As the global population ages and national health expenditures grow, we believe that uncovering the key individual and cross-cutting themes of Starfield’s 4Cs will allow PHC systems to take stock of their capacities and learn from the case studies of other countries. Therefore, our study explores how Starfield’s 4Cs of PHC can empower health systems to manage the uptick in chronic disease loads through trialled and tested policies of countries reviewed.

## Methodology

Our study includes case studies gathered from 19 countries by integrating findings from a comprehensive literature review and a qualitative study. We selected these countries as they represent a spectrum of income levels and diversity in health systems and PHC landscapes (see table 1). All countries also face an ageing population but performed differently during the height of the COVID-19 pandemic, offering us a comprehensive view of PHC policies.

**Table 1:** Description of key statistics from 19 selected country case studies

	Country	GDP per capita 2021	UHC index 2021	UHC Index 2019	Health expenditure per capita 2016	Health expenditure per capita 2021	Population size in thousands 2022	NCDs burden (DALYs) 2019	NCDs burden (DALYs) 2021
Africa	Rwanda	822	49	47	128	180	13 777	23 143	23 452
	Ghana	2422	48	46	163	248	33 476	24 893	24 433
Asia	Nepal	1229	54	50	159	228	30 548	23 767	23 882
	Vietnam	3760	68	69	403	536	98 187	20 327	20 195
	Indonesia	4334	55	56	317	483	275 501	24 499	24 476
	Thailand	7071	82	82	639	967	71 697	18 095	18 181
	China	12 618	81	81	664	1033	1 412 175	18 880	18 785
	Singapore	79 601	89	88	3855	6453	5637	12 255	12 052
Europe	Poland	18 050	82	82	1295	1639	37 562	19 470	19 431
	Spain	30 489	85	85	3341	4368	47 615	15 658	15 541
	United Kingdom	46 870	88	81	4321	6266	66 971	17 764	17 552
	Austria	53 518	85	84	5477	6505	9043	16,33	15 900
	Norway	93 073	87	86	6271	9163	5457	15 455	15 366
	Slovenia	29 331	84	85	2873	2873	2109	15 649	15 245
North/ South Americas	Colombia	6183	80	80	1052	15 42	51 874	16.723	16 833
	Mexico	10 363	75	74	1089	1190	127 504	21 538	21 827
	Argentina	10 651	79	78	1998	2323	46 325	19 710	18 884
	Uruguay	17 734	82	83	1954	2335	3423	19 974	20 076
	USA	71 056	86	85	9600	12 474	333 288	21 385	21 762

All numbers are rounded to the nearest whole number and in US$ where appropriate. Gross domestic product per capita from 2021 (World Bank). Universal Health Coverage Index 2019 and 2021 (WHO). Health expenditure per capita and population in 2016 (World Bank). Population size in thousands in 2022 (World Bank). NCDs burden is determined by calculating disability-adjusted life years per 100 000 individuals from NCD causes in 2019. To allow comparisons between countries and over time, this metric is age-standardised (Institute of Health Metrics and Evaluation (IHME), Global Burden of Disease).

NCDnon-communicable chronic disease

PHC is operationalised in our study as a system that interfaces with all levels of care, addressing the populations’ needs across their life course and encompassing preventive, promotive, reactive and rehabilitative care. We used Starfield’s 4Cs of PHC as a starting point to identify the key themes for each ‘C’ regarding chronic disease management in these countries. In this paper, we also provide snapshots (in the boxes below) through key illustrations of how PHC employs each of the ‘Cs’ to manage NCD in the host country while acknowledging that this might not be fully reflective of the entire PHC system in the country due to the heterogeneity of the health system, unequal fiscal resources across subregions and differences in governance among other factors.

Briefly, C1: ‘*Comprehensive care*’, expands on the availability of a wide range of services in primary care to cater to a spectrum of health conditions. In the context of NCD management, comprehensive care would allow for early prevention and diagnosis of conditions, provide timely treatment and promote health and wellness. C2: ‘*First contact of care*’ refers to care first sought from the primary care provider (ie, services must be accessible and used by the population each time new health or medical need arises) to establish preliminary connection to healthcare services, enable cost-effective gatekeeping, to reduce overuse of tertiary healthcare services. C3: ‘*Coordination of care*’, discusses the deliberate organisation and navigation of patient care activities across various care providers. This is necessary considering the numerous facets of NCD management that require care beyond PHC. C4: ‘*Continuity of care*’ describes the longitudinal use of a regular care provider for constant monitoring and follow-up. This would allow for the fostering of a provider-patient relationship over time, improving the uptake of preventive care and adherence to treatment. Since 1992, when these 4Cs were first articulated, they remain highly relevant to managing chronic conditions and are used as cornerstones for PHC innovations and reforms.^12 13^

### Available data sources

We systematically gathered data from two different data sources, including a literature review from electronic databases and secondary data from interviews with experts in the country’s health systems and financing.

First, studies were screened and selected from PubMed, Google Scholar and government websites to obtain data on chronic disease management in the selected countries. To achieve this, we employed standard search terms that included PHC, care coordination, continuity of care, comprehensive care, first contact of care, NCDs, chronic diseases, health policy and country names, including alternative spellings and synonyms to consolidate data from all 19 countries. Boolean operators and truncators varied depending on the database searched. All academic studies and articles (policy briefs and technical reports) from January 2015 to June 2023 were considered for inclusion as the case studies aimed to offer the most relevant PHC climate of the 19 countries prepandemic and postpandemic, where radical changes occurred for most countries. No exclusion criteria were applied in order to capture how primary care is linked to other care levels and other services beyond its own ambit.

Second, we also employed secondary data (not from this current study) to help fill the gaps in the desk reviews. These data were derived from semi-structured, in-depth interviews with 61 key stakeholders in the fields of health systems focusing on questions on the country’s health systems and financing, including a primary care element.^14^

Furthermore, primary care experts, who are health system researchers and part of the research team, and are well-informed about each of the 19 countries, were provided with a standardised data collection template to gather the data sets. This also facilitated the translation of data not originally in English. After data collection, these country experts would ensure the validity and accuracy of the data collected from literature searches and qualitative interviews before data analysis began.

### Data extraction and management

Data extraction was conducted using a customised data extraction form in Microsoft Excel. The extraction format was refined afterwards to ensure feasibility as well as standardisation as data collection was implemented by country experts and researchers.

### Data synthesis and analysis

The compiled and consolidated data sets were analysed inductively using thematic analysis and deductively using Starfield’s ‘4C’ framework. In brief, the data sets were coded based on the salient aspects of our research question and organised into themes, namely the ‘4Cs’, so as to map the codes and themes to the domains featured in Starfield’s original framework. Thereafter, final themes and subthemes were agreed on among all the authors after multiple rounds of iterative feedback and thematic saturation was reached when no new themes emerged. The key themes that emerged are italicised in the ‘Results’ and ‘Discussion’ sections.

## Results

**Figure 1:**
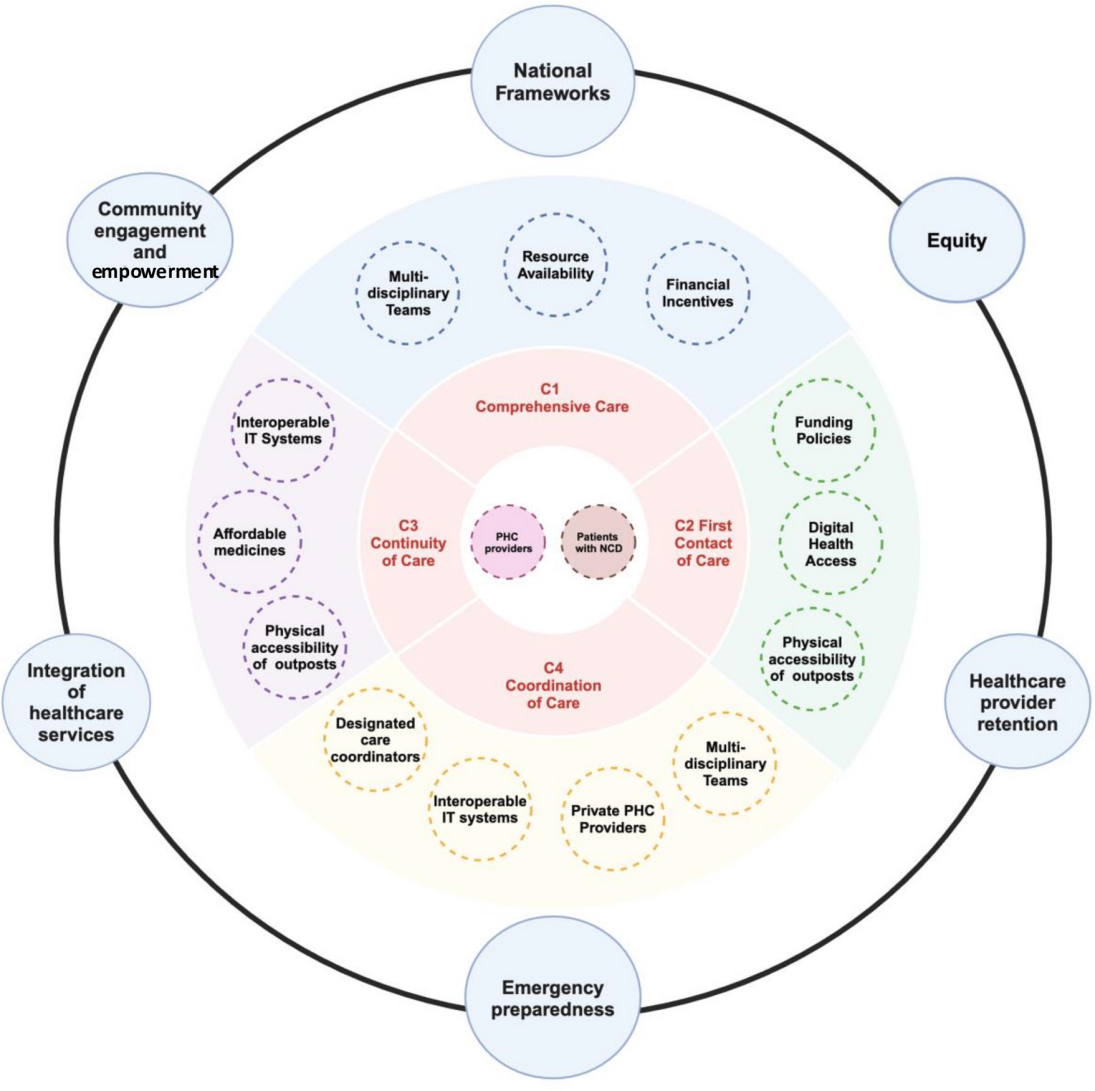
Conceptual framework of themes and subthemes based on Starfield’s 4Cs for non-communicable chronic disease (NCD) management at the primary healthcare (PHC) level. IT, information technology.

Based on our review of PHC systems in 19 countries, we identified several subthemes over the Starfield’s 4Cs of PHC that contributed to successful NCD management at the PHC level, integrating them into a cohesive framework (figure 1). Anchoring the 4Cs as the core of the framework, we expanded on the core themes by integrating the relevant subthemes around the 4Cs and crucial elements that span across all the 4Cs and subthemes at the outer rim. This was performed as an iterative process of back-and-forth discussions with all authors to ensure the subthemes and overarching concepts (outer rim) fit relevantly into the framework, keeping patients and providers at the nexus PHC delivery. In the following sections, we explain our findings according to this framework and provide case studies illustrated concisely in the boxes below.

### C1: Comprehensive care

The countries reviewed had shown that PHC providers offered a wide range of services for NCD management. We found that these services span the continuum of care, from screening and immunisation to social prescribing and other forms of ancillary services. In addition, for most LMICs, their PHC providers also offer maternal and child healthcare services, serving as a one-stop avenue for families to meet their health needs. A few factors made the delivery of these additional services possible. These include ***national frameworks*** such as national immunisation schedules, financial reimbursement per capita for screening services and additional human resources. Certain comprehensive services, such as vaccinations, diabetic foot screening and diet counselling, were also provided for by non-general practitioners (GPs), including nurses and dietitians who are employed at the PHC level. Broadly, most PHC providers work in ***multidisciplinary teams that expand the spectrum of services beyond what a traditional solo practitioner could offer, expanding the range of healthcare needs satisfied at the PHC level****.* We also found that affordable ***healthcare financing*** was addressed through financial incentives ***from both national and private stakeholders, tailored to providers’needs to drive service expansion***. Providing financial means to attain ***sufficient medical and screening resources***, medications, equipment and increase competencies of a multidisciplinary team, enable the provision of more medically relevant services.

In LMICs, ***task shifting*** is also often seen, as non-medical or paramedical personnel, including volunteers, offer medically relevant services such as health education and engagement, expanding the impact of a multidisciplinary team in boosting preventive care, screening rates and adherence to treatment at the PHC level. Members of such multidisciplinary teams also share tasks while adopting additional roles in the non-clinical environments. However, the lack of fiscal resources has hindered the offering of free medical services at the point of care, ***which affects equitable healthcare access to comprehensive services, more apparently seen in LMICs***. Unfortunately, in most LMICs, ***disparities in service provision was noted***, particularly in less developed and rural areas of the country, due to medical resource and manpower constraints, geopolitical considerations and physical inaccessibility, illustrating intracountry heterogeneity in terms of resource allocation. The case studies are summarised in box 1.

Box 1 **Case studies of comprehensive services offered for non-communicable disease (NCD) management**

**Multidisciplinary teams**

Singapore: A reform called HealthierSG was implemented in mid-2023, whereby general practitioners (GPs) are empowered (financially and manpower-wise in the form of a multidisciplinary team) to offer social prescribing services by linking their patients to community partners that offer physical activity, well-being and socioeconomic support functions to keep populations healthy through the life course.

USA: In New York, community health workers provided educational lessons to patients with chronic conditions or at risk for chronic conditions to help them manage their conditions, as tertiary hospitals were inundated with COVID-19 cases during the pandemic. These workers also made home visits, conducted wellness checks over the phone, helped people enrol in online patient portals and prepared them for telehealth appointments.

Slovenia: Services are provided by multiprofile teams, which evidence indicates are essential for effectively addressing the healthcare needs of a population primarily affected by NCDs. The introduction of reference family medicine clinics in 2011 represented a significant organisational and substantive enhancement in GP practices, facilitated by the integration of a qualified nurse into the team for half of the working hours. The primary objective is to provide comprehensive, team-based patient care, focusing on preventive activities, patient empowerment, health education and management of selected NCDs. Protocols for the care and management of the NCDs were developed, ensuring coordination among all healthcare levels, including family medicine specialists and clinical specialists.

Thailand: Village health workers (VHWs) work with nurses and public health officers to support NCD services at community level. They deliver health education services, advocacy and basic screening services (eg, diabetes, hypertension) to patients with NCD. Although most PHC centres have no full-time doctors, within the district health system, doctors from district hospitals and from different specialities, also rotate to provide clinical services to patients with NCD at PHC centres where NCD cases are high, for a few days per week.

**Resource availability**

Colombia: PHC services are expanded to include preventive, curative and rehabilitative care that extends beyond the traditional health facilities to reach underserved communities (although financially constrained). For example, the provision of viral hepatitis medicines is focused on in the contributory and subsidised health insurance schemes and there is emphasis on the delivery of sexual and reproductive healthcare to poor communities, the migrant Venezuelan population, transgender individuals and people with disabilities. However, some public healthcare providers are limited by low quality of healthcare provision, underfunding and limited resources to adapt these into equity-oriented health services. Furthermore, PHC services focused on obesity, hypertension, diabetes, mental health disorders and long COVID-19 are not fully developed in the private and public healthcare services settings.

Vietnam: Commune health centres (CHCs) in Vietnam offer a suite of health services. However, these services are not distributed equally across provinces. Specifically, CHCs offer limited medical examination and treatment and might not have a full range of drugs to treat common NCDs and, in particular, mental health diseases due to a lack of medical supplies and trained medical personnel in more rural provinces.

Nepal: Pregnant women can obtain antenatal care for free and are provided with incentives for antenatal care visits and transportation to deliver in health facilities, while basic necessities such as clothing are provided for the mother and newborn at PHC facilities. A comprehensive suite of services, such as family planning and counselling, are also provided at no cost.

**Healthcare financing**

Uruguay:

NCD care is included in the guaranteed services plan, Plan Integral de Atención a la Salud (PIAS), offered by the comprehensive providers of the National Integrated Health System. These services encompass everything from outpatient consultations to second-level and third-level care, including intensive care units. Medication coverage is provided through the National Therapeutic Formulary, which includes several high-cost medications. The PIAS is guaranteed by the network of services that integrates primary healthcare and hospital services within the context of the country’s primary healthcare framework.

Poland: There has been a recent expansion of the range of forms of remuneration for the work of GPs, nurses and midwives. The basis for remuneration in PHC care has long been the capitation rate. At the present time, it is enriched by various additional methods of payment for services, such as fee for service, monthly flat rate, entrusted budget (lump sum), motivation/incentive allowance and entrusted budget for additional NCD services offered at the primary care level.

### C2: First contact of care

Most countries have introduced PHC providers as the gatekeepers to the health system. Gatekeeping is typically seen as an avenue to redirect patients from overusing hospitals for uncomplicated medical needs that could have been treated at the primary care level. Countries may adopt various policies to reinforce gatekeeping mechanisms based on the architecture of their health systems. Countries with a strong presence of payer(s) with a considerable market share of beneficiaries may direct patients to seek care at the primary care level and require them to seek referral letters from their PHC providers before they can access more specialised care at the secondary and tertiary levels. In this setting, NCD care is often included in the standard benefit packages offered by the contracted PHC providers. Thus, payers are often interested in reducing the burden of hospitals by encouraging patients to talk to their GPs about early detection and disease management at the primary care level, preventing serious complications that might cost payers a lot more in the future. In other settings with a strong presence of private PHC providers, governments may have limited power to mandate strong gatekeeping roles to GPs, and patients already enjoy unrestricted access to any care. Therefore, governments may introduce monetary incentives to encourage patients to seek NCD care at private PHC providers (eg, GP clinics or polyclinics) rather than accessing specialist care or tertiary hospitals. Thus, ***appropriate health financing policies influence first-contact feasibility for both PHC providers and the population seeking medical care***.

PHC providers in all countries were reported to use teleconsultation tools, making them the ***accessible digital gateway into the health system***. Postpandemic, some countries continue to cover NCD virtual visits (that were traditionally not covered by subsidies or national health insurance prepandemic), lowering the financial barriers to receiving digital PHC services and ***improving digital health access***. Additionally, some countries have established mobile clinics or health posts (usually closer to the communities) at the PHC level to ***improve physical accessibility*** so that PHC providers can become an accessible first contact point. The case studies are summarised in box 2.

Box 2 **Case studies of primary healthcare (PHC) being the first contact point for non-communicable chronic disease (NCD) management**

**Healthcare financing**

Indonesia: Under the JKN Programme (national health insurance scheme), each participant must be registered in a PHC facility that is empanelled in the BPJS Kesehatan (Social Security Agency on Health) systems to obtain any medical service, except in emergency situations. Medical cases that are within the scope of competence of the PHC as a first-level health facility must be completely resolved at PHC except for selected cases that BPJS Kesehatan and local health authorities have agreed on having them treated at the secondary and tertiary hospitals.

Poland: PHC doctors in Poland are the first contact of care who have the power to decide who can receive publicly reimbursed specialised care. The range of services provided by PHC providers has also expanded through the Entrusted Budget so that PHC providers can purchase additional resources and get reimbursed for the additional services offered to patients. Thus, patients can seek PHC providers for more complex issues before going to higher levels of care.

UK: The National Health Service (NHS) in England provides care, free at the point of use, while general practitioners (GPs) remain the primary contact for any NCD-related symptoms pre-acute and post-acute hospital admission. While technically all GPs are privately owned, 76% of them receive incomes from NHS funding via Clinical Commissioning Groups (replaced with Integrated Care Systems in 2022), mandating the provision of universally covered PHC services at GP clinics.

Colombia: Patients’ health insurance schemes dictate the speed of referral to specialist care. In addition to empanelment and registration, there are a few other forms of gatekeeping that are used in the Colombian PHC system. For example, some healthcare providers require patients to obtain a pre-authorisation from purchasers before seeing a specialist. However, this has led to delays in care, inefficient processes and increased medical complications.

Norway: The GP card, known as the ‘Frikort’, allows patients to access PHC services without direct payment for certain medical treatments once they reach a specific threshold of out-of-pocket expenses. Once a patient has paid a certain amount in co-payments for health services within a calendar year, they can apply for a Frikort, which exempts them from further co-payments for the remainder of that year. This system helps to ensure that healthcare remains accessible to all citizens, particularly those with lower incomes or NCDs, who will in turn be able to make PHC their first touch point with the health system.

**Physical accessibility of outposts**

Rwanda: Empanelment is typically based on geographical proximity. The health posts serve as an interface between community health workers and health centres. Patients are assigned to the PHC facility located nearest to their residence, ensuring convenient physical access to care. However, a lack of adequate medicines, equipment and staffing created long waiting times and insufficient healthcare at these PHC facilities despite free medical coverage for the population. Patients will then have to walk long distances to reach higher levels of care.

Norway: Out-of-hours, emergency primary care (EPC) services serve as the initial point of contact with the Norwegian healthcare system for all residents. These services are designed to ensure that individuals have access to necessary medical care at any time, particularly during evenings, weekends and holidays when regular clinics may be closed. Each EPC service is strategically organised to cover one or more municipalities, allowing for flexibility based on local healthcare priorities and needs. This arrangement enhances the accessibility of medical services for the population and ensures that resources are allocated efficiently to meet the specific demands of different communities.

**Digital health access**

Singapore: Technology such as teleconferencing and mobile apps allowed SingHealth Polyclinics (SHP) to further enhance patient care while reducing the number of clinic visits. In 2010, TeleCare services were launched. With TeleCare, patients monitor their own blood pressure at home and nurses will call them to review their conditions making this the continuous first touch base for their medical follow-up. Based on their readings and review, patients would be advised to collect their new supply of medication or see a general practitioner for further assessment.

Mexico: Information technology for health has been supported in Mexico mainly by the Mexican Social Security Institute (IMSS) through electronic medical records, the private sector through apps and Ministry of Health (MoH) hospitals and state-level health services through telehealth. PHC providers rapidly adopted telehealth applications to provide teleconsultations for patients with NCDs and for home monitoring of patients who were discharged from hospitals. 45 000 teleconsultations are provided at MoH facilities in 15 states, supporting mostly mental health and internal medicine. In the highly rural state of Oaxaca, a total of 19 telehealth-equipped peripheral units and one central hospital offered 53 teleconferences and nearly 5000 consultations across five specialties, with maternal health a priority.

Austria: Teleconsultations are becoming more widespread in Austria, particularly benefiting the rural and underserved populations, providing a range of services from primary care (eg, mental healthcare, general consultations, prescription refills, health advice), chronic disease management, specialist and postoperative care, making digitalised PHC an accessible first port of call when medical problems arise. Teleconsultations are generally financed by the Austrian healthcare system, with patients paying the same co-payments as for in-person consultations, although there are some differences among private PHC providers.

Slovenia: A key component of the Slovene strategy for PHC is the use of information and communication technology, enhancing the functionality of e-consultations. This improves accessibility for patients, particularly those in rural or underserved areas, and streamlines the workflow for healthcare professionals. Additionally, the strategy envisions the formation of interdisciplinary groups (comprising providers from different care levels) that address complex cases through videoconferencing.

### C3: continuity of care

In most countries reviewed, there are models of care and programmes that facilitate the transition of stable patients with NCDs from the hospital to the community for management by PHC providers. These patients with NCDs typically have manageable chronic conditions such as hypertension, diabetes and high blood pressure. As the countries’ populations continue to age, such programmes integrating PHC with higher levels of care will enable patients with chronic conditions to be managed by a PHC provider in the community for the long term and increasingly ***through digital means***. This approach will free up tertiary resources for more complex conditions. Furthermore, key enablers, such as ***interoperable IT systems that allow providers at all care levels to viewpatientdata and appropriate payment packages across care providers*** are cornerstones. Moreover, having medicines available at PHC levels that are ***heavily subsidised or covered by health insurance*** helps to further anchor patients requiring long-term chronic care in the community. However, inequitable resource distribution or shortages can fragment care, as seen in most LMICs reviewed. PHC systems are often underfunded, and patients do not receive the services they need, which leads to loss of follow-up and worsening of their health conditions. In some resource-constrained countries, community outposts or commune centres that offer basic medical services were also seen to promote care continuity by ***improving physical accessibility***. The case studies are summarised in box 3.

Box 3 **Case studies of primary healthcare (PHC) promoting continuity of care for non-communicable chronic disease (NCD) management**

**Healthcare financing**

UK: A pay-for-performance component is implemented for general practitioners working in NHS England, and they are rewarded based on the fulfilment of certain criteria, which includes a minimum number of follow-up appointments and responsibility to monitor patients over the time course, which encourages continuity of care. Long-term NCD care provided to the population remains free of cost.

Singapore: A capitation model for provider payment will be rolled out in mid-2023 in phases. This aims to promote care continuity as providers are incentivised to care for the individual patient throughout the life course as they are reimbursed per capita on an annual basis. However, these funds will only be disbursed after PHC providers have followed up with the patients and completed the necessary ancillary services, such as diabetic retinopathy and diabetic foot screening, required to show that continuation of care had been provided for NCDs.

Spain: The main sources of public financing for healthcare in Spain are from social security contributions, which are mandatory payments made by employers, employees and taxes. These contributions are used to fund the Spanish National Health System (Sistema Nacional de Salud), which provides portable healthcare fee coverage to all Spanish citizens, regardless of which catchment area they see the healthcare provider at, ensuring patients receives continuous follow-up care when required without fear of inability to pay.

**Interoperable information technology systems**

Argentina: The country implemented a digital health strategy to have interoperable health information systems that allow the implementation of electronic medical records in the health system (including public, private and social security), improvement of connectivity and training staff to manage the new medical records system. Although implementation has progressed slowly, this strategy seeks to promote continuity of care when patients leave the acute hospitals for management by PHC providers in the community as patients’ medical history can be seen at all levels of care and even in the private sector.

Nepal: NepalEHR, although only implemented in a few districts, has given PHC providers an opportunity to access and share data critical for continuity and coordination of care. Specifically, shared access to electronic medical records between facilities and community health workers helps to provide facility-based and community-based providers with continuity of data that enables them to appropriately identify threats to population health and longitudinally track patient outcomes for more effective, person-centred service delivery and informational, managerial and relational continuity of care at the PHC level.

Austria: It has one of the most digitally advanced healthcare systems in Europe, with a universal electronic health record and strong linkages between different data sources. It is one of only six countries in Europe with the operational and technical capacity to use electronic health records to generate information, and one of 10 that can link hospital and mortality data.

**Physical accessibility of community outposts**

Uruguay: Doctors and nurses make periodic visits to the residences of patients with NCDs who were discharged from hospitals with a focus on providing continuous care for people in Long-Stay Etablishments for Older Persons (ELEPEM), one of the most vulnerable groups, considering age) and the provision of medicines to these people in the community. The services and medicines provided to socioeconomically vulnerable groups are free of cost.

China: The enhancement in services offered and resources provided at rural village health clinics was employed to shift the demand of basic health services to the primary care level by offering more preventive, promotive and rehabilitative services. Despite this, sociocultural norms had shaped the demand for healthcare services towards large tertiary hospitals and also declining residential-health facility distance when seeking higher care institutions.

Rwanda: Health posts are intermediary primary care facilities located at the community level. They provide relatively comprehensive primary care services to communities and are located within a reasonable walking distance from people’s homes. They also serve as an interface between health centres and community health workers. These health posts offer basic longitudinal care to patients with ongoing medical conditions to patients who otherwise might have not returned for follow-up due to physical distance from their providers at higher levels of care.

Vietnam: Commune health centres (CHCs) form the grassroots public healthcare system in rural Vietnam, where two-thirds of the country’s people live. The centres provide basic preventive care along with referrals to public hospitals and continual management postdischarge for NCD conditions when tertiary hospitals where overwhelmed with infected cases during the pandemic.

Ghana: The Community-based Health Planning and Services (CHPS) approach to healthcare provision remains the major strategy being adopted for the provision of PHC services. CHPS engages the local communities to create health services that are in most demand by the end users, which include follow-up antenatal care, chronic disease management and continuous infectious disease intervention that promotes followup care for local village populations across the timeline.

### C4: Coordinated care

In a few countries, there are reports of the ***presence of designated care coordinators*** who are responsible for linking patients to other levels of care and also to other sectors, both within and beyond the healthcare system. Notably, ***multidisciplinaryteams and collaborations across sectors and levels of care*** promoted coordinated care throughout the patient journey, highlighting the need for buy-in at all levels of care and also at the national and regional policymaking levels. Only PHC providers in certain countries reviewed had designated care coordinators to facilitate the movement of patients beyond the PHC clinic setting. Some health systems also ***engage private PHC providers to complement their public PHC facilities, with government financing*** to support the care for patients with NCDs in the community after discharge from public hospitals. However, protocols need to be clearly delineated and contractual agreements adhered to for such collaborative models to work. Additionally, the proliferation of ***interoperable IT systems and a workforce trained in providing coordinated care*** can ensure a safer and more efficient transition of patients across different levels and sectors of care. Unfortunately, widespread usage of interoperable IT systems remains a challenge in most lower-income countries as IT systems remain disjointed, and many providers continue to rely on a paper-and-pen system. The case studies are summarised in box 4.

Box 4 **Case studies of care coordination for non-communicable chronic disease (NCD) management**

**Multidisciplinary care coordinators**

Singapore: In primary care networks (PCN), case managers and nurses are hired to link patients to ancillary services, which might not be provided at the PHC clinics themselves. The clinics are also reimbursed for patients successfully linked to ancillary and follow-up services on a per capita per annum basis. All public sector healthcare entities must be on the National Electronic Health Record (NEHR) system that allows patient data to flow seamlessly across different care levels aiding the coordinated movement of patients between care settings.

Poland: Care coordinators are established at PHC levels, but the new model of financing—Entrusted Budget—which offers more NCD services at the PHC level, lacks legal provisions as there are no specified tasks or additional funding for coordination.

UK: Mental health coordinators (who are usually nurses or social workers) were available in most PCN and would refer patients who were suffering from mental health conditions or predisposed to mental health illnesses. These mental health coordinators will coordinate the services offered to these patients between general practitioners and mental health professionals.

Vietnam: Local PHC collaborations were initiated through multiple methods, such as a memorandum of understanding (MOU) between the Provincial or District Health Office and the local authorities (under the Ministry of Interior) or MOUs between the Provincial and District Health Office, District Health Coordinating Committee, local nursing colleges and private sectors. Some district or provincial hospitals have established NCD committees comprising a multidisciplinary team coordinating patient journeys.

Thailand: At district level, NCD case managers, who are mostly nurses, are trained to oversee patient’s outcomes, coordinate care for patients with NCD and give consultation to the multidisciplinary team (eg, family doctors, nutritionists, public health officers, volunteer health workers) to provide care for patients with NCD to ensure updated patient progress is integrated across all care interfaces.

**Interoperable information technology systems**

Rwanda: limited interoperability of government systems, where many health facilities work in isolation, and the electronic medical record (EMR) system used in some health facilities still provides multiple patient identification numbers within one or many health facilities, while they still rely on paper-based systems. A lack of information flow hinders the coordination of patient movement across care entities.

Singapore: All public sector healthcare entities must be on the NEHR system that allows patient data to flow seamlessly across different care levels.

Nepal: In most of the districts, data generated are in manual formats at PHC facilities and sent to the Department of Health Services using Health Management Information System forms as monthly reports. This manual data management system often causes complication in coordination.

UK: The Health and Social Care Information System (HSCS IS) is an ongoing project, created to unify and streamline healthcare data management across the country. This ambitious project aims to create a single, centralised repository of patient information, accessible to authorised healthcare professionals, enabling seamless care coordination and improved clinical decision-making. The HSCS IS is designed to be interoperable with existing healthcare systems, enabling seamless data exchange and integration with third-party applications. This interoperability will facilitate the exchange of patient information across various care settings. It is also committed to engaging patients in the development and implementation of the electronic patient record system, to ensure that patients are also engaged in the maintenance of sharing of their medical records.

Poland: The e-health system is the bedrock of the digital ecosystem of medical services in Poland, enabling the collection, processing and sharing of digital resources about patients’ medical events and electronic medical records indexes across all levels of care. It also integrates applications for patients to access their EMRs, telehealth facilities and request prescription renewals. To encourage adoption of this system by all care providers across geographical areas, national funding is provided to develop local cabinet software and support PHC facilities in building their own local repositories to enable them to provide access to medical records. Private PHC providers are also obligated to access the e-health system. This system is in its transitional stage and will be implemented in phases at the national level.

Norway: Norwegian vendors are leveraging openEHR as a solid technical and semantic foundation for developing their systems. In PHC, PatientSky is promoting coordinated care, which improves patient outcomes and boosts the overall efficiency of the healthcare system. With its innovative e-health solutions (EHR, Patient Administration System, Telemedicine Services, Patient Engagement Tools, Data Analytics, Interoperability and Chronic Disease Management Tools), they are establishing a standard for how technology can enhance health management and patient care through improved data sharing and interoperability.

## Discussion

While no universal template can be applied to all countries, enhanced implementations of the 4Cs can promise to yield substantial benefits to patients and broader populations. The strategies that manifest the 4Cs must be tailored to become fit-for-purpose based on the climate of individual countries’ PHC systems and how they situate themselves in the broader health system. To illustrate, we have offered case studies of 19 countries from across the continents depicting how each PHC system attempted to uphold the 4Cs in the context of NCD management.

As the global population ages and the burden of NCDs is ever-increasing, PHC will play a more significant role in managing the uptick in patients with NCD. Centrally, our results point to multiple ways the factors manifesting the 4Cs overlap extensively. In this discussion, we will elaborate on how this is so.

### C1: Comprehensive care

Considering the multifactorial nature of patients’ NCD experience, ***comprehensive care*** should ideally integrate various facets of healthcare, including PHC, tertiary care and public health. Providing a comprehensive range of services at the PHC level enables patients to receive the services that they would normally receive at higher levels of care and much earlier, to prevent disease progression. This is often achieved by having a *multidisciplinary team and adequate financing packages that reimburse PHC providers for additional services offered.* Furthermore, ***task shifting*** by training or allowing non-clinically trained personnel to take on clinically relevant tasks with physician oversight was seen as a means to offer a wider array of services at the PHC level.^15^ In addition, VHWs were observed as a key conduit to achieve this, particularly in LMICs, where clinical staff are in short supply in more rural areas, and care can also be provided in a more ***culturally appropriate and equitable manner***.^16^ In addition, adequate ***fiscal resources will need to flow*** to these providers to purchase medical equipment and medicines and reimburse personnel to expand the list of NCD services offered.^17^

### C2: First contact of care

***The first contact of care*** through national policies that make PHC providers the front door to the health system can ensure that tertiary care resources are preserved only for the patients that need them the most. PHC can act as a gatekeeper to identify populations at risk of developing NCD complications and initiate a holistic treatment strategy involving multiple health workers, including community health workers, nurses and community pharmacists. Having PHC operate through multidisciplinary teams comprising medical and paramedical personnel further creates the window of opportunity for the population to seek care at lower levels for more disease conditions.^18^ The degree of gatekeeping that redirects patients from specialised care to primary care will vary from one country to another, and the success of such efforts will predicate on both provider and population factors. Notably, factors such as ***affordable health financing and improving physical and digital accessibility*** reiterate PHC as the front door to the health system for NCD care for the population. To become the first touch point, providers should also be able to provide the necessary services and be well-equipped to conduct their expanded scope of services safely.^19^

Healthcare innovations for NCDs are increasingly taking the form of digital health transformations. Promoting teleconsultations for NCD care will improve accessibility and serve as a means to alleviate the lack of colocation of PHC providers.^20^ In the countries reviewed, the government reimbursed teleconsultations for providers offering digitalised services and patients seeking remote consultations, displaying the feasibility and adoption of virtual consultations for NCDs. This improves the accessibility for patients by making PHC providers a convenient and continuous first port of call going forward. The usage of digital technology for healthcare was found to be highly acceptable for patients and providers in the countries reviewed and, coupled with medicine delivery services, protected providers and patients from nosocomial transmissions during the height of the COVID-19 pandemic.^21^ Hence, shifting digital toolkits for NCD management and covering the costs of these remote services by factoring them into existing insurance structures warrants further exploration. In many countries, ***physical accessibility of outposts*** remains a crucial hurdle to integrating current and future patients with NCD into the healthcare system and in providing preventive care. There are also limitations to building a patient-doctor relationship in order to understand patients’ psychosocial, financial and family needs through a virtual platform. However, information technology could potentially be leveraged to improve digital health access for vulnerable populations and to serve as the ‘entry point’ into establishing first contact of care.

### C3: Continuity of care

Continuity refers to coherent, linked care among patients, families, communities and providers across lifetimes. It involves understanding individuals’ contexts with longitudinal clinical information and using this knowledge to build trusting relationships over time.^22^ Key levers include ***interoperable information systems*** that allow patient information to flow seamlessly across providers within and between levels of care for information and, in turn, achieve care continuity through the patient journey while allowing for the measuring of PHC performance indicators.^23^ ***Equitable financing*** is a crucial component for patients to continue seeking care at the PHC level, and this is often seen through the removal of co-payments. ***Lowering or eliminating financial barriers*** can promote PHC as the first point of contact and ensure continued engagement with the health system due to low barriers to entry and follow-up care. Therefore, PHC can offer equitable basic healthcare by discarding co-payments and ensuring that PHC services are universal and equitable.^24^ This can also pave the way for making medical services and medications more affordable, which is crucial given the multifaceted nature of NCD disease management, progression and long-term care needs. Improving affordability would be a fundamental step in improving adherence, encouraging individuals to take charge of their health and entrenching their continued engagement with the healthcare system.

### C4: Coordinated care

#### Care coordination

Care coordination is a tenet that ensures PHC providers are well-linked to other essential services across levels of care and beyond the healthcare system. In most countries, there were no designated care coordinators. Instead, nurses or social workers usually double up as care coordinators, often viewed as a form of task-shifting when PHC providers operate in a ***multidisciplinaryteam***.^25^ However, to ensure smooth care coordination in a resource-poor setting that necessitates task shifting, investment into training and the establishment of designated care workflows is paramount to ensure optimal patient care and to avoid role strain and overload. This is particularly crucial in an ever-evolving healthcare landscape with an increasing NCD burden. In most countries, patients (usually stable) can transition across levels of care and between facilities. The liaison between care entities is facilitated by having an ***interoperable IT system*** that allows providers at all care entities to have a bird’s eye view of a patient’s medical history, augmenting the coordination of care and enabling providers to transfer patient data across care echelons, especially when patients are discharged from tertiary to the PHC level.^26^ This will be particularly salient in health systems that employ ***private PHC providers*** to co-manage patients with NCD with specialists in the hospital setting.^27^ However, we must acknowledge that digitalised platforms may not be accessible to all populations, and resources, including IT infrastructure, might not be available for widespread implementation in certain settings.^28^

By breaking down the 4Cs into subthemes, the framework provides PHC providers, policymakers and researchers with greater clarity on the components of each C, enabling targeted and actionable changes. This iteratively developed conceptual framework, derived from global country examples, helps countries prioritise subaspects, identify specific limitations and understand the necessary infrastructure and financing required to optimise their existing healthcare systems. Overall, the conceptual framework shows the extent of collaboration required across PHC providers and multiple levels of care in synergistically leveraging on multiple dimensions to successfully meet the needs of PHC providers and patients with NCDs, with them at the nexus.

While most aspects of the PHC systems that were reviewed has shown some high-level semblance of Starfield’s 4Cs of PHC, there is much left to be done. We need to leverage this window of opportunity that presents itself post-COVID-19 and ride on the wave that places PHC at the core of a health system by recognising that PHC can confer multifold benefits through protecting population health writ large during peacetime and health crises.^29^ In the paragraphs below, we outline various arguments that extend beyond the scope of PHC and into the broader health system, as shown by the overarching themes that encapsulate the 4Cs of the PHC system. This is illustrated in the outer rim of our framework.

From a centralised perspective, ***national frameworks and policies*** are fundamental towards pivoting the strategic direction of policy development and implementation. They emphasise PHC’s role in addressing the 4Cs towards robust NCD management, restructuring various resources and integrating multiple levels of care digitally and physically. National health organisations need to ensure transparency and accountability in the provision of fiscal incentives and financial co-payments. The overarching governance and leadership buttresses the foundation on which subsequent levels of the 4Cs and their subthemes can be built.

The recent COVID-19 pandemic has shown how healthcare emergencies can rapidly destabilise health systems, highlighting the importance of ***emergency preparedness***. To prevent the neglect of non-emergent conditions such as NCDs in future healthcare emergencies, countries explored in this study have since established emergency responder funds, taskforces, workflows and clinical guidelines to ensure rapid response to such emergencies while prioritising existing healthcare demands. Immunisation and infectious disease control, information systems activities, development of best practice guidelines, quality assurance and evaluation strengthen existing healthcare systems to better serve current patients while ensuring preparedness to weather future healthcare emergencies such as the COVID-19 pandemic. Yet, the pandemic also contributed to innovative strategies, such as the advent of digital applications and the rapid training and deployment of village volunteers and social workers to overcome geographical barriers in the delivery of care.^30 31^ In the non-emergent setting, these strategies should continue to be leveraged for the delivery of NCD care while increasing their competencies in emergency response.

The COVID-19 pandemic has also shown the severe impact of underinvesting in healthcare workers. In reality, every country faces a shortage of healthcare workers, and the shortfall is only projected to worsen.^32^ In LMICs, health inequities at the PHC level are exacerbated by chronic underfunding and healthcare workers preferring to work in non-rural settings, further straining existing PHC facilities.^33^ Thus, governments can introduce task shifting, harnessing non-clinical staff such as community health workers to provide basic NCD-related services. This must be coupled with the necessary training and accreditation for PHC providers to manage cases normally managed at higher care levels or by specialists.^15^ More importantly, steps need to be expeditiously undertaken to prevent healthcare worker burnout, improve remuneration and protect the well-being and welfare of healthcare workers to ensure ***healthcare provider retention*** and competent service delivery.^34^ Several countries have implemented policies to improve manpower retention, such as special allowances for those working in rural areas, fixed-term rotation of health staff at districts and communes, development of open feedback and communication channels and employee recognition and rewards. Furthermore, the planning and forecasting of human resources are included in such policies, particularly in the face of the ageing population and emergency preparedness. Such efforts would pave the way towards ensuring that the PHC sector has adequate human resources to provide quality NCD services and respond to shocks to the system. But for such aspirations to materialise, sustainable investments that are accountable and equitable need to flow to frontline primary care facilities while employing cost-effective strategic purchasing tools to ensure medical resources, both human and material, are adequate to anchor PHC as the epicentre of the health system.^14 35^

Bidirectional policy development and implementation are predicated on ***community engagement and empowerment***. Social participation in implementing certain PHC policies which have been co-created can help make these policies more durable and palatable in the long run.^36^ This can be done by a multidisciplinary team at the primary care level that may also comprise volunteers or community health workers, as seen in the context of Thailand, to serve as a link between the health system and the community. This can also serve as a means to cultivate trust between system and population. Furthermore, chronic disease management and screening services should be integrated to serve current patients with NCD and to connect patients with high NCD risk to the healthcare system, such that they receive preventive healthcare and lifestyle advice much earlier. In countries facing manpower constraints and geographical inaccessibility, preventive health and public health ventures such as community engagement and empowerment can be conducted through digital means. Such measures should be integrated into the larger healthcare landscape and national frameworks to ensure equitable delivery of healthcare services and to ensure continued NCD care in the event of future healthcare emergencies.

Crucially, even as we place increased emphasis on PHC for NCD management, secondary and tertiary care must not be neglected.^37^ Higher levels of care are still required to manage more complex and severe cases of NCDs. In a health system, PHC and higher levels of care need to work in concert through ***integrated models of service delivery***, which also includes unified and commensurate financial mechanisms for all providers to deliver ***equitable*** and optimal care for patients with NCD in a collaborative fashion.^38^ The integration can also involve co-engineered care models between providers across care interfaces and the community so that integrated programmes assimilate the expectations of all stakeholders and reduce any unintended consequences.

The various themes and subthemes illustrated in this study provide countries with a multidimensional outlook in boosting the comprehensiveness and competencies of their healthcare systems, showing the interconnections between the 4Cs and their respective subthemes and the necessity of factoring in the concepts in the outer rim of our framework in ensuring integration and coordination of PHC with the larger healthcare system. To ensure the sustainability and resilience of NCD management in the face of uncertainties, all themes and subthemes need to be evaluated and factored in parallel into future policy decision-making.

To generalise from these convergent streams of thought and action, continuing disparities in health conditions between and within countries must be addressed by employing a whole-of-systems approach that focuses on equity and sustainability, such that individual and collective action at all levels can be relevant and mutually reinforcing. We have also summarised the priority recommendations based on the key themes and subthemes in the conceptual framework in box 5.

Box 5 **Policy recommendations**

**C1: Comprehensive care**

*Expandmultidisciplinary teams*

Integrate clinical and paraclinical personnel including volunteers and community health workers as key stakeholders in collaborative primary, tertiary and public healthcare teams to enable delivery of comprehensive care services across care interfaces.

*Expandmultidisciplinary teams*

Empower non-clinically trained personnel including village health workers situated in primary healthcare settings to address manpower constraints and to enhance delivery of culturally appropriate care in rural settings while reducing overall strain and burnout to existing clinical human resources.

*Increase financial support*

Provide increased fiscal resources to enhance the purchase of medical equipment and medicines to expand range of non-communicable chronic disease (NCD) services offered at the primary healthcare (PHC) level. Providers also need to be commensurately reimbursed for services provided while out-of-pocket costs to population seeking PHC services reduced or removed.

*Address rural inequities*

Improve infrastructure, implementing sound digital services to overcome physical inaccessibility and incentivising healthcare professionals to serve in rural settings to lower barriers to physical entry for rural populations can ensure a wider range of services are offered to far-reaching communities.

**C2: First contact of care**

*Enhancegatekeeping mechanisms*

Implementing national frameworks and policies that promote PHC providers as the first touch point in the health system prior to up-triaging when appropriate. In parallel, governments will also need to boost capabilities of PHC services through the development of integrated models of care delivery with higher levels of care and digital health platforms that make it easier for population to access PHC providers.

*Digital health accessibility*

Enhance teleconsultation capabilities for NCD care to improve digital health access for vulnerable populations, acting as the initial touchpoint for healthcare. Covering the cost of digitalised health services while improving health and information technology (IT) literacy of the population will reduce barriers to using this digital doorway to the health system.

*Establish mobile clinics*

Mobile clinics that complement the services offered at PHC outposts can aid in addressing physical inaccessibility to basic PHC services offered at health outposts to integrate current and future NCD patients into existing healthcare systems to bridge the gap for those living far away from main cities and hence hospitals.

**C3: Continuity of care**

*DevelopinteroperableITsystems*

Improve flow of patient information across different healthcare facilities and enable the measurement of PHC performance indicators for policy evaluation purposes. Measuring PHC performance indicators will enable policymakers to identify gaps and provide targeted redress based on the objective indicator outcomes.

*Eliminate financial barriers*

Remove copayments to reduce barriers to entry for both physical and digital consultations, improving access to basic healthcare in a universal and equitable fashion by putting the most left behind first will ensure more equitable distribution and uptake of basic health services.

*Promote longitudinal care*

Enhance affordability of care to improve adherence and health empowerment in view of the progressive nature of NCDs. Regular and consistent follow-up with PHC providers will reduce complications of NCDs and improve not just the lifespan but health span of the patients.

**C4: Coordinated care**

*Introduce designated carecoordinators*

Train designated care coordinators and establish care workflows to ensure proper transition across care facilities and prevent role strain and overload incurred by current task-shifting borne by existing clinical staff.

*Strengthen public-private collaboration*

Establish partnerships between public and private PHC providers and higher care providers to co-manage patients with NCD through building trust between sectors and establishing co-created and fiscally viable contractual agreements that are patient and provider centred.

*Invest in it infrastructure*

Digitalising the health space to implement interoperable IT platforms that enable providers at all care levels an overarching view of patients’ current and evolving health needs will equip care providers to safely move patients between facilities in view of the complex healthcare needs of patients with NCD.

**Overarching recommendations**

*Adopt national frameworks*

Pivoting strategic directions of policy development and implementation for adequate restructuring of available resources and integration of multiple levels of care through evidence-based policies co-engineered through bidirectional community engagement and all-stakeholder buy-in is essential for policy longevity.

*Prioritising equity*

Ensuring an equity lens in all health policies approach to position the most left behind first by removing financial barriers to health services and having policies and health services meet the most vulnerable where they are.

*Invest in emergency preparedness*

Establishing emergency responder funds, taskforces, clinical guidelines to respond swiftly to medical emergencies while ensuring the continuation of high-quality delivery of non-emergent NCD care when shocks hit the system, and equipping PHC providers with the necessary skills and resources to protect the health of the population.

*Supporthealthcare workers*

Introducing task-shifting, improve remuneration and protect well-being and welfare of healthcare workers to improve healthcare provider retention, reduce burnout and promote quality services is imperative in the face of a global shortfall of healthcare workers.

*Engage communities*

Co-creation of PHC policies to encourage social participation and cultivate trust between populations and healthcare systems is critical in developing PHC policies that are palatable and durable for all parties involved. This also helps reduce unintended consequences of policies implemented.

*Leveragedigital innovations*

Optimising digital means for community engagement and education in overall health to counter manpower constraints and geographical inaccessibility as the world moves towards more digitally empowered health systems. Digital literacy and proliferation of digital tools need to come in parallel to ensure equitable access for all populations.

*Foster integration across care levels*

Establishing unified and commensurate financial mechanisms through co-engineered care models between providers across care interfaces and community stakeholders to deliver team-based and collaborative care, often needed to manage more complex patients with NCD.

This paper also acknowledges that the subsets of themes that emerge are not exhaustible and the major subthemes can percolate across the 4Cs of primary care. In addition, the subthemes are constrained by the data collected from the respective countries and might not be generalisable to all healthcare systems. As there are only case studies for two African countries, there remains an under-representation of analysis from that continent that requires further analysis going forward. The next step will be to deploy this framework and evaluate how primary care systems perform in the aspects derived in this study.

## Conclusion

The implementation of an integrated PHC system for robust NCD management is critical under the auspices of the imminent increase in NCD burden, ageing populations and rising healthcare costs against the backdrop of the post-COVID-19 pandemic. Our study demonstrates how various themes of Starfield’s 4Cs can be harnessed by PHC systems to provide comprehensive care by expanding competencies and range of NCD management services, to enable first contact of care through digital health access and outposts, to augment care continuity through various methods to encourage patient retention and reception and to coordinate care between multiple levels of the healthcare system, as NCD needs continue to evolve. Although there are existing constraints in resource availability, physical accessibility and fiscal unavailability, prioritisation of PHC development for NCD management can be harnessed as a long-term cost-effective strategy to provide equitable healthcare.

## Data Availability

Data are available on reasonable request.
